# Evaluation of Knowledge and Self-Awareness of Polish Soldiers Regarding Exposure to Chemical and Physical Factors in Their Place of Service/Work

**DOI:** 10.3390/healthcare12040450

**Published:** 2024-02-09

**Authors:** Ewelina Ejchman-Pac, Małgorzata Rzepka, Małgorzata Gierlach, Paweł Szymański, Magdalena Zawadzka

**Affiliations:** 1 Department of Hygiene and Epidemiology, Military Institute of Hygiene and Epidemiology, 01-163 Warsaw, Poland; ewelina.e.pac@wihe.pl; 2 Prevention and Treatment Team, Department of Organization of the Health Care System, Department of Military Health Service, 00-911 Warsaw, Poland; malrzepka@mon.gov.pl; 3 Military Unit 2305, 04-520 Warsaw, Poland; mbgierlach@interia.pl; 4 Department of Radiobiology and Radiation Protection, Military Institute of Hygiene and Epidemiology, 01-163 Warsaw, Poland; pawel.szymanski@umed.lodz.pl; 5 Department of Pharmaceutical Chemistry, Drug Analyses and Radiopharmacy, Medical University of Lodz, 90-419 Lodz, Poland; 6 Department of Epidemiology and Public Health, Medical University of Lodz, 90-419 Lodz, Poland

**Keywords:** soldiers, exposure, knowledge level, chemical factors, physical factors

## Abstract

Introduction: Soldiers constitute a professional group carrying out their duties in variable, often challenging environmental conditions, including harmful and burdensome ones. Materials and Methods: This study was conducted on a nationwide sample of 1331 soldiers. The research tool was an anonymous questionnaire comprising 48 questions. Descriptive statistics were used to describe the characteristics of the studied group. The chi-square test was employed to examine the relationship between variables. A 95% confidence interval was adopted, with a significance level of *p* = 0.05. Results: One in four soldiers work with low and one in five with moderate exposure to harmful chemical factors. Almost 10% of respondents lack knowledge about the types of chemical factors present during their service. One in five soldiers work with low and one in eight with moderate exposure to ionizing radiation. Approximately 5% of survey participants lack knowledge about the types of physical factors. One in three soldiers are unaware of the carcinogenic and mutagenic effects of the aforementioned factors. Conclusions: The systematic enhancement of knowledge and awareness among army members will help minimize the consequences of exposure to harmful conditions.

## 1. Introduction

Soldiers represent a professional group performing their duties in variable and often challenging environmental conditions, including harmful and burdensome ones. The main sources of threats during soldiers’ service can be identified during intense training, especially shooting exercises (noise), prolonged field training, extreme exercises (e.g., diving, parachute jumps), handling military equipment and vehicles, exposure to explosive materials, lubricants, and participation in missions in various climatic conditions beyond the state borders. Consequently, these individuals are particularly exposed to various dangerous factors, including carcinogenic, mutagenic, and reprotoxic (chemical and physical) elements. Therefore, the identification of harmful and burdensome factors during service, the periodic measurement and sampling of these factors in the workplace, as well as the provision of individual and collective protective measures by the employer, are crucial. Another equally important issue is the continuous training of soldiers regarding existing hazards and ways to reduce their negative effects, as prolonged exposure to carcinogenic, mutagenic, and reprotoxic factors can have serious consequences for the health of military personnel (occupational diseases, long-term sick leave, disability, hereditary mutations, harmful effects on fertility, and ultimately death). Among the most frequently occurring physical hazards and harmful or burdensome factors in the military service environment are hazards related to the operation of computer monitors, noise, and electromagnetic fields, while among chemical factors, exposure to extraction gasoline, naphtha, xylene, and toluene predominates. Other commonly identified hazards during service include inorganic dust containing crystalline silica, work at heights up to 3 m, work requiring psychomotor skills, and hazards associated with positions of responsibility and decision-making.

According to the Regulation of the Minister of National Defense of 2 March 2023, *on the leave of professional soldiers*, service positions where the performance of professional military service is harmful to health are positions where duties are performed for at least half of the applicable service time under conditions harmful to health, and four levels of harmfulness are indicated. The first level of harmfulness results, among other aspects, from exposure to the action of dust that does not cause the fibrosis of lung tissue and exposure to non-cumulative toxic substances. The next level of harmfulness is the result of exposure to the action of dust causing the fibrosis of lung tissue, cumulative toxic substances, and exposure to noise. The third level of harmfulness is determined by exposure to the action of high-frequency electromagnetic fields from 0.1 MHz to 300,000 MHz in a danger zone. Exposure to dust or aerosols of soluble salts of heavy metals has been classified as the fourth level of harmfulness [1].

Exposure to the above-mentioned factors is highest among soldiers performing daily tasks related to the production, destruction, storage, and transportation of explosive, flammable, and self-igniting materials, as well as in the storage, distribution, and neutralization of hazardous substances with chemical properties. Furthermore, the impact of harmful chemical factors on soldiers’ work environment, compared to the civilian environment, is multiplied due to the intense training soldiers undergo during field exercises (the inhalation of dust and fumes from armored vehicles or during chemical defense training using battlefield simulation measures) [2].

According to data collected in the reports on occupational safety and health (service), the Department of Social Affairs of the Ministry of National Defence reports that in 2022, 10,475 professional soldiers, constituting 8.85% of the total number of professional soldiers in the Polish Armed Forces (according to the publication of the Central Statistical Office “Small Statistical Yearbook of Poland 2023”, the Polish Armed Forces at the end of 2022 had 118,300 professional soldiers), served under conditions threatening to health, including carcinogenic factors [3]. Considering the constant exposure of soldiers and the concern for their current and future health, efforts should be made to minimize the effects of exposure to harmful and burdensome factors to the necessary minimum.

When analyzing the degree of exposure to harmful factors among military personnel, it is worth noting that this issue also affects other professional groups. In their work, Kauppinen et al. state that over 32 million (23%) workers employed in various sectors of the economy in 15 selected European Union countries are exposed to carcinogenic factors in the workplace. The analysis shows that the most common carcinogenic substances include, among others, solar radiation (9.1 million people), tobacco smoke (7.5 million people), diesel engine exhaust (3.0 million), radon (2.7 million), wood dust (2.6 million), benzene (1.4 million), formaldehyde, and polycyclic aromatic hydrocarbons (PAH) [4].

To address these challenges, a survey was conducted among 1331 professional soldiers regarding their awareness of exposure to carcinogenic chemical and physical factors, as well as their knowledge level about these substances. The results of this survey are the subject of this publication.

## 2. Materials and Methods

This study, the results of which are presented here, was conducted on a nationwide sample of soldiers. The Military Center for Civic Education—Military Social Research Bureau (WBBS) was responsible for drawing the sample and its implementation. The study was conducted in September and October 2022. The research tool was an online survey (CAWI). It consisted of 38 substantive questions and 10 socio-demographic questions. Respondents were reached through the WBBS research panel. A total of 1331 individuals were surveyed. After completing the study, the WBBS provided raw research results in EXCEL format.

Consent to conduct the study was obtained from the Bioethics Committee at the Military Medical Chamber in Warsaw; Approval Code: 11/23; Approval Date: 19 May 2023.

The aim of the study was to assess the awareness of exposure to carcinogenic chemical and physical agents and to assess the level of knowledge about these substances.

The following research hypotheses were formulated:

**H_0_:**  *The level of knowledge and self-awareness of soldiers regarding exposure to chemical and physical factors does not depend on socio-demographic variables*.

**H_1_:**  *The level of knowledge and self-awareness of soldiers regarding exposure to chemical and physical factors depends on socio-demographic variables*.

## 3. Software and Statistical Analysis

Due to the quantitative nature of the study (survey), the GNU PSPP 2.0.0 program was used for result analysis. Procedures used in the analysis included the following:

- Frequency analysis (single-variable analysis);
- Correlation analysis (using contingency tables and statistics);
- Correlation analysis using the Spearman coefficient.

A 95% confidence interval was adopted for statistical analysis, and a significance level of *p* = 0.05 was set, indicating statistically significant results for levels of *p* < 0.05.

## 4. Characteristics of Respondents

Out of 1331 respondents, 60 did not provide their age. Therefore, the basis for calculations for this characteristic was 1271 participants. In total, 94.3% of participants were male. A significant percentage of surveyed soldiers (46.7%) were between the ages of 31 and 40, with 40.8% falling in the 41–50 age range. A total of 6.7% of respondents were under 30 years old. At the same time, it should be noted that at the extreme end, i.e., in the group over 50 years old, just over 5.9% of respondents were recorded. The average age was 40.06 years. The largest group among respondents (mode) consisted of those aged 35, i.e., 6.53% of participants.

### 4.1. Education

Over 2.0% had vocational education, more than half had secondary education (53.5%), and 44.2% had higher education.

### 4.2. Military Rank and Service Tenure

Among the respondents, 24.9% were privates, just over 53.4% were non-commissioned officers, 9.3% were junior officers, and 12.4% were senior officers. Respondents had professional service tenures ranging from 3 to 40 years. The largest group among the respondents consisted of soldiers with a fifteen-year service tenure, i.e., 11.3% of respondents. The average service tenure was 16.8 years. A detailed analysis of the collected data revealed that the dominant group (48.9%) consisted of individuals with service tenures between 11 and 20 years. A total of 50 respondents did not answer this question. Respondents held staff positions—19.3%; command positions—21.9%; specialist positions—28%. Other specialist positions were held by 15.3%, and the remaining 15.2% described their positions as “other.”

### 4.3. Participation in Foreign Deployments or Missions

Over half of the respondents, 59.4%, did not travel abroad or participate in missions as part of their official duties, while the remaining 40.6% declared participation in foreign deployments and missions.

## 5. Results

### 5.1. Chemical Factors

#### 5.1.1. Self-Awareness of Exposure to Harmful Chemical Factors

Nearly one-fourth of the respondents are not exposed to harmful chemical factors. One-fourth of those surveyed work in low-exposure environments, while one-fifth work in environments with moderate exposure to the aforementioned factors. Approximately 6% of individuals indicated a presence of very high exposure to harmful chemical factors, with around 9% experiencing high exposure. Statistically significant differences were found between the variables: respondents’ age, education, military service corps, military service tenure, and respondents’ official positions vs. exposure to harmful chemical factors. Detailed analyses regarding self-awareness of exposure to harmful chemical substances based on gender, age, education, military service corps, military service tenure, and respondents’ official positions are presented in Table 1.

#### 5.1.2. Assessment of Knowledge Level Regarding Types of Chemical Factors during Service

Almost 90% of respondents possess knowledge regarding the types of chemical factors encountered during their service, with half of them having full knowledge and the remainder having partial knowledge. One in ten survey participants lack awareness on the subject. Statistically significant differences were found between the variables: respondents’ age, length of military service, and their level of knowledge about the types of chemical agents during service. Detailed analyses regarding the knowledge level of types of chemical factors during service based on gender, age, education, military service corps, military service tenure, and respondents’ official positions are presented in Table 2.

### 5.2. Physical Factors

#### 5.2.1. Self-Awareness of Exposure to Ionizing Radiation

More than half of the respondents are not exposed to ionizing radiation. One-fifth of the respondents work with low and one-eighth with moderate exposure to the aforementioned factors. Approximately 6% of individuals indicated high exposure to ionizing radiation, while around 4% reported very high exposure. Statistical significance was demonstrated between the following variables: age, military service corps, tenure of military service, military rank vs. self-awareness of exposure to ionizing radiation. Detailed analyses regarding self-awareness of exposure to ionizing radiation based on gender, age, education, military service corps, length of military service, and respondents’ official positions are presented in Table 3.

#### 5.2.2. Assessment of the Level of Knowledge Regarding the Physical Factors Occurring during Service

Almost every respondent has knowledge about the physical factors occurring during their service. More than one-fifth of the respondents have complete knowledge in this area, and one-fourth have partial knowledge. Less than 5% of the study participants have no knowledge on the subject. No statistical significance was demonstrated between the studied socio-demographic variables and the level of knowledge about the physical factors occurring during service. Detailed analyses regarding the level of knowledge about the physical factors occurring during service based on gender, age, education, military service corps, length of military service, and respondents’ official positions are presented in Table 4.

#### 5.2.3. Evaluation of Awareness of Carcinogenic and Mutagenic Effects of Physical Factors

More than half of the respondents are aware of the carcinogenic and mutagenic effects of physical factors. One-third of the participants lack knowledge in this area. Statistical significance was demonstrated between the following variables: gender, official position vs. respondents’ awareness of carcinogenic and mutagenic effects. Detailed analyses regarding awareness of the carcinogenic and mutagenic effects of physical factors based on the gender, age, education, military service corps, length of military service, and job position of respondents are presented in Table 5.

## 6. Discussion

Occupational diseases and injuries, as well as the service performed, result in significant losses for those affected, employers, and society as a whole. The percentage of many work-related diseases can be substantial, although this does not always imply that the disease is also recognized as an occupational illness. It is estimated that 3.2–4.6% of all cancer-related deaths are attributed to occupational exposure [5]. Various methods have been developed to assess the economic costs for businesses and society. Disability-adjusted life years (DALYs) are often used as an approximate measure of the burden of occupational and work-related diseases [6].

The strategic framework of the European Commission (EC) on occupational safety and health for the years 2021–2027 defines key priorities and actions to improve the safety and health of workers, taking into account rapid changes in the economy, demographics, and work patterns. It focuses on three key priorities: anticipating and managing changes in the context of ecological, digital, and demographic transformations; improving accident and illness prevention at work and striving for a “Vision Zero” approach to work-related deaths; enhancing readiness to respond to current and future health crises [7,8].

The success of these “frameworks” relies on effective law enforcement, social dialog, funding, awareness-raising, and data collection. Aligning with the strategic framework of the EC, it was decided to assess the awareness among professional soldiers of the Polish Armed Forces regarding their personal exposure to harmful physical and chemical factors. As is known, every type of work is associated with risks, but military service has its unique aspects not present in the civilian environment. Serving in variable climatic conditions exposes military personnel to harmful chemical factors such as sand, dust, toxic air, and water pollutants. Research conducted by Sanders et al. in the U.S. Forces population participating in the Iraqi Freedom and Enduring Freedom operations from 2003 to 2004 showed that the most common health issues among military mission participants were respiratory diseases resulting from significant exposure to chemical factors. Among the surveyed 15,000 soldiers, 69% reported respiratory diseases, with 17% requiring medical care [9].

In our own research on exposure to chemical factors, almost every fourth respondent is not exposed to harmful chemical factors. Approximately 27% of individuals showed low exposure to chemical factors, 8.8% showed high exposure, and 5.5% exhibited very high exposure. Similar studies were conducted in the Norwegian Navy. Respondents were asked about exposure to various chemical factors, including exhaust fumes, oil and gasoline (skin contact), and lead. Low exposure was reported by 18–25.9% of respondents, high exposure by 6.1–8.8%, and very high exposure to the mentioned chemical factors by 3.1–5.2% of individuals. The results obtained by Moet et al. significantly correlate with our own findings. The characteristics of participants and research methods were also similar, with both studies utilizing an online survey. The difference lies in the relationship between variables: the gender of respondents and exposure to harmful chemical factors. Our study did not show statistical significance between these variables, whereas in the Norwegian Navy population, women overall reported lower exposure to various harmful factors compared to men [10].

Important for military personnel is exposure to ionizing radiation, a physical factor with potential health implications. Many artificial sources of ionizing radiation in the military are similar to those found in industries, hospitals, nuclear power plants, and research centers. Soldiers are exposed to radiation during military operations and combat training (the testing of nuclear weapons, cobalt bombs, or artificial radioactive isotopes). It is worth noting that a large amount of military equipment contains radioactive material, which, if damaged, can be a source of harmful radiation. In the U.S. Army, approximately 70,000 individuals (2% of the total personnel) are monitored annually for ionizing radiation exposure. This monitored group provides an excellent database for epidemiological research [11].

According to our research, about 4% of respondents showed very high exposure to ionizing radiation. A detailed analysis regarding exposure to this factor in correlation with socio-demographic variables revealed a statistically significant dependence between the professional corps of the respondents and the assessment of exposure to radiation. No statistical significance was found between gender, education, and the assessment of exposure to ionizing radiation.

Similar studies were conducted by the Military Institute of Radiobiology and Radiation Protection among U.S. military personnel in response to concerns about ionizing radiation (fear of receiving even low doses of radiation can significantly impact soldiers’ combat readiness in military operations). No relationship was found between the assessment of exposure to ionizing radiation and gender, but exploratory studies showed a connection between education and professional corps and knowledge about the level of exposure to ionizing radiation [12].

In our own study assessing the level of knowledge about the types of harmful physical factors present during service, approximately 5% of respondents lack knowledge in this area, and almost 41% have only partial awareness of the threat. This correlates with cross-sectional studies conducted on a group of 409 soldiers in the army of Saudi Arabia. This questionnaire-based study, assessing the level of awareness of noise-related hazards, the most common harmful factor during service, showed that over half of the participants (54.3%) were unaware of the impact and consequences of noise exposure. As a result, few soldiers used hearing protection measures [13]. This underscores the importance of training and meetings with specialists, contributing to increased awareness of hazards and protection against harmful physical and chemical factors.

As indicated above, approximately 5% of respondents lack knowledge about harmful conditions in which they serve, and almost 41% of respondents have only partial awareness of the threat. Extrapolating the results to the entire population of soldiers in the Polish Armed Forces, 524 soldiers exposed to harmful and dangerous factors lack knowledge about the threats, and 4295 soldiers have only partial knowledge in this area. Therefore, it must be assumed that due to low self-awareness regarding existing threats, they do not apply or selectively apply individual and collective preventive measures against the adverse health effects of threats in their place of service. Consequently, it can be expected that among this group, the occurrence of health disorders related to the specific nature of their service will be significantly more frequent than in individuals using full recommended protection. For example, prolonged exposure to noise can contribute to hearing loss, tinnitus, and consequently, chronic stress and depression. Another example is prolonged exposure to diesel fumes, which irritate mucous membranes; may cause nausea; and directly contribute to the risk of lung cancer, heart disease, and respiratory system disorders.

These and other examples of health consequences related to exposure to hazardous factors in the place of service are, of course, related to the dose and duration of exposure and the initial health status, but the aim of this work is to raise awareness of the need to reduce the number of soldiers unaware of existing threats, thus protecting their health and lives.

## 7. Conclusions

1. Over 60% of respondents are aware of exposure to chemical agents at their place of work.
2. In the study group, approximately 11% of soldiers have no knowledge and over 45% have insufficient knowledge regarding the types of chemical factors occurring during service.
3. Over 40% of respondents are aware of radiation exposure at the workplace.
4. Only every second soldier surveyed has full knowledge of exposure to ionizing radiation.
5. More than 30% of people in the study group are not aware of the carcinogenic and mutagenic effects of physical factors.

Systematic improvements in knowledge and awareness among military members will help minimize the effects of being in harmful conditions, translating into the quality of service and readiness for action. This undoubtedly contributes to enhancing the defense potential of the country in situations of threat or conflict.

## Figures and Tables

**Table 1:** Self-awareness of exposure to harmful chemical factors due to socio-demographic variables.

		Harmful Chemical Factors				
		No Exposure	Low	Moderate	High	Very High Exposure
Gender	Male	38.0%	27.0%	20.6%	8.8%	5.6%
	Female	44.4%	25.0%	18.1%	8.3%	4.2%
	Total	38.3%	26.9%	20.4%	8.8%	5.5%
Statistics		Chi^2^ = 1.329, df = 4, *p* = 0.856				
Age	25–30 years	22.6%	27.4%	35.7%	11.9%	2.4%
	31–40 years	29.7%	29.2%	24.9%	9.6%	6.7%
	41–50 years	48.4%	25.4%	13.6%	7.7%	4.9%
	51–60 years	58.1%	17.6%	16.2%	4.1%	4.1%
	Total	38.4%	26.8%	20.6%	8.7%	5.5%
Statistics		Chi^2^ = 75.539, df = 12, *p* < 0.05				
Education	Vocational	30.8%	34.6%	23.1%	0.0%	11.5%
	Secondary	32.3%	26.9%	23.7%	11.3%	5.9%
	Higher	46.0%	26.5%	16.5%	6.2%	4.8%
	Total	38.4%	26.9%	20.4%	8.8%	5.5%
Statistics		Chi^2^ = 37.699, df = 8, *p* < 0.05				
Military service corps	Professional Private	23.3%	29.4%	26.5%	12.9%	8.7%
	Sergeant	37.8%	26.6%	20.9%	9.1%	5.7%
	Junior Officer	47.1%	25.2%	21.8%	3.4%	2.5%
	Senior Officer	66.0%	24.4%	5.8%	3.2%	0.6%
	Total	38.4%	26.9%	20.5%	8.8%	5.5%
Statistics		Chi^2^ = 107.922, df = 12, *p* < 0.05				
Military service tenure	3–10 years	27.6%	30.7%	26.1%	8.8%	6.9%
	11–20 years	33.4%	26.9%	22.8%	10.5%	6.4%
	21–30 years	55.2%	25.5%	11.6%	4.8%	2.9%
	31–40 years	50.8%	19.7%	18.0%	6.6%	4.9%
	≥41 years	33.3%	20.0%	20.0%	26.7%	0.0%
	Total	38.4%	26.9%	20.4%	8.8%	5.5%
Statistics		Chi^2^ = 77.620, df = 16, *p* < 0.05				

**Table 2:** Assessment of the level of knowledge regarding types of chemical factors during service due to socio-demographic variables.

		Knowledge Level Regarding Types of Chemical Factors during Service		
		No, I Do Not Have Such Knowledge	Yes, I Have Partial Knowledge	Yes, I Have Full Knowledge
Gender	Male	11.1%	45.6%	43.3%
	Female	8.1%	45.9%	45.9%
	Total	10.9%	45.6%	43.4%
Statistics		Chi^2^ = 0.690, df = 2, *p* = 0.708		
Age	25–30 years	9.5%	53.6%	36.9%
	31–40 years	9.1%	50.9%	40.0%
	41–50 years	12.0%	40.6%	47.4%
	51–60 years	18.9%	33.8%	47.3%
	Total	10.9%	45.9%	43.2%
Statistics		Chi^2^ = 21.330, df = 6, *p* = 0.002		
Education	Vocational	5.3%	57.9%	36.8%
	Secondary	10.2%	46.1%	43.7%
	Higher	12.0%	44.6%	43.4%
	Total	11.0%	45.6%	43.5%
Statistics		Chi^2^ = 2.466, df = 4, *p* = 0.651		
Military service corps	Professional Private	10.6%	47.0%	42.4%
	Sergeant	10.1%	47.0%	42.9%
	Junior Officer	14.9%	43.8%	41.3%
	Senior Officer	12.5%	38.1%	49.4%
	Total	11.0%	45.6%	43.4%
Statistics		Chi^2^ = 6.536, df = 6, *p* = 0.366		
Military service tenure	3–10 years	8.7%	49.8%	41.5%
	11–20 years	9.8%	48.2%	41.9%
	21–30 years	13.4%	39.8%	46.9%
	31–40 years	18.0%	34.4%	47.5%
	Total	10.9%	45.7%	43.4%
Statistics		Chi^2^ = 14.177, df = 6, *p* = 0.028		
Military rank	Specialized—Technical, Equipment Operation, Operator, etc.	9.7%	49.4%	40.9%
	Other Specialized	8.8%	43.8%	47.4%
	Other	12.8%	44.4%	42.9%
	Staff	16.5%	43.1%	40.3%
	Other Staff Command (including Deputy, Command Assistant)	8.2%	44.8%	47.0%
	Total	11.0%	45.6%	43.4%
Statistics		Chi^2^ = 15.303, df = 8, *p* = 0.054		

**Table 3:** Self-awareness of exposure to ionizing radiation due to socio-demographic variables.

		Ionizing Radiation				
		No Exposure	Low	Moderate	High	Very High Exposure
Gender	Male	5.3%	21.0%	12.2%	5.7%	3.9%
	Female	64.4%	16.4%	12.3%	2.7%	4.1%
	Total	57.7%	20.7%	12.2%	5.5%	3.9%
Statistics		Chi^2^ = 2.356, df = 4, *p* = 0.671				
Age	25–30 years	44.0%	21.4%	22.6%	7.1%	4.8%
	31–40 years	50.3%	24.9%	13.9%	6.3%	4.6%
	41–50 years	68.0%	15.0%	9.7%	4.3%	3.0%
	51–60 years	60.0%	26.7%	6.7%	4.0%	2.7%
	Total	57.5%	20.8%	12.4%	5.4%	3.9%
Statistics		Chi^2^ = 49.270, df = 12, *p* < 0.05				
Education	Vocational	52.0%	24.0%	8.0%	8.0%	8.0%
	Secondary	57.9%	21.3%	11.5%	5.7%	3.6%
	Higher	57.6%	19.8%	13.3%	5.1%	4.2%
	Total	57.7%	20.7%	12.2%	5.5%	4.0%
Statistics		Chi^2^ = 3.636, df = 8, *p* = 0.888				
Military service corps	Professional Private	48.7%	24.4%	14.7%	7.4%	4.8%
	Sergeant	59.3%	20.1%	11.8%	5.4%	3.4%
	Junior Officer	57.1%	21.0%	14.3%	3.4%	4.2%
	Senior Officer	69.2%	16.0%	7.7%	3.2%	3.8%
	Total	57.7%	20.7%	12.2%	5.4%	3.9%
Statistics		Chi^2^ = 22.418, df = 12, *p* = 0.033				
Military service tenure	3–10 years	51.0%	21.3%	16.3%	6.5%	4.9%
	11–20 years	54.3%	22.5%	13.1%	6.2%	3.9%
	21–30 years	71.5%	14.6%	7.8%	3.2%	2.9%
	31–40 years	51.6%	32.3%	8.1%	4.8%	3.2%
	≥41 years	52.9%	17.6%	11.8%	5.9%	11.8%
	Total	57.7%	20.7%	12.2%	5.5%	4.0%
Statistics		Chi^2^ = 42.837, df = 16, *p* < 0.05				
Military rank	Specialized—Technical, Equipment Operation, Operator, etc.	48.4%	26.9%	14.0%	6.8%	3.9%
	Other Specialized	58.1%	23.0%	11.0%	3.1%	4.7%
	Other	62.5%	18.2%	16.1%	2.6%	0.5%
	Staff	50.6%	21.1%	12.1%	9.8%	6.5%
	Other Staff Command (including Deputy, Command Assistant)	75.3%	13.0%	8.4%	1.3%	2.1%
	Total	57.8%	20.7%	12.3%	5.4%	3.9%
Statistics		Chi^2^ = 80.398, df = 16, *p* < 0.05				

**Table 4:** Assessment of the level of knowledge about the physical factors occurring during service due to socio-demographic variables.

		Knowledge Level Regarding Types of Ionizing Radiation		
		No, I Do Not Have Such Knowledge	Yes, I Have Partial Knowledge	Yes, I Have Full Knowledge
Gender	Male	4.5%	41.5%	53.9%
	Female	4.1%	45.2%	50.7%
	Total	4.5%	41.7%	53.8%
Statistics		Chi^2^ = 0.385, df = 2, *p* = 0.825		
Age	25–30 years	4.7%	42.4%	52.9%
	31–40 years	4.2%	45.2%	50.6%
	41–50 years	4.5%	38.2%	57.4%
	51–60 years	4.0%	42.7%	53.3%
	Total	4.3%	42.0%	53.7%
Statistics		Chi^2^ = 5.671, df = 6, *p* = 0.461		
Education	Vocational	0.0%	47.4%	52.6%
	Secondary	4.2%	43.4%	52.4%
	Higher	5.1%	39.5%	55.4%
	Total	4.6%	41.7%	53.7%
Statistics		Chi^2^ = 3.336, df = 4, *p* = 0.503		
Military service corps	Professional Private	3.4%	43.6%	53.0%
	Sergeant	4.9%	42.6%	52.5%
	Junior Officer	6.6%	36.4%	57.0%
	Senior Officer	3.8%	37.7%	58.5%
	Total	4.6%	41.7%	53.8%
Statistics		Chi^2^ = 5.436, df = 6, *p* = 0.489		
Military service tenure	3–10 years	4.5%	41.8%	53.7%
	11–20 years	4.2%	43.9%	51.9%
	21–30 years	5.9%	37.0%	57.1%
	31–40 years	3.2%	41.9%	54.8%
	Total	4.6%	41.6%	53.8%
Statistics		Chi^2^ = 5.100, df = 6, *p* = 0.531		
Military rank	Specialized—Technical, Equipment Operation, Operator, etc.	3.8%	43.6%	52.6%
	Other Specialized	3.6%	43.6%	52.8%
	Other	5.6%	44.2%	50.3%
	Staff	5.6%	42.2%	52.2%
	Other Staff Command (including Deputy, Command Assistant)	4.6%	35.9%	59.5%
	Total	4.6%	41.7%	53.7%
Statistics		Chi^2^ = 7.365, df = 8, *p* = 0.498		

**Table 5:** Evaluation of awareness of carcinogenic and mutagenic effects of physical factors due to socio-demographic variables.

		Awareness of Carcinogenic and Mutagenic Effects of Physical Factors	
		No	Yes
Gender	Male	35.6%	64.4%
	Female	18.9%	81.1%
	Total	34.6%	65.4%
Statistics		Chi^2^ = 8.576, df = 1, *p* = 0.003	
Age	25–30 years	29.4%	70.6%
	31–40 years	34.5%	65.5%
	41–50 years	35.6%	64.4%
	51–60 years	33.3%	66.7%
	Total	34.5%	65.5%
Statistics		Chi^2^ = 1.319, df = 3, *p* = 0.725	
Education	Vocational	40.9%	59.1%
	Secondary	35.4%	64.6%
	Higher	33.7%	66.3%
	Total	34.7%	65.3%
Statistics		Chi^2^ = 0.799, df = 2, *p* = 0.671	
Military service corps	Professional Private	32.3%	67.7%
	Sergeant	36.6%	63.4%
	Junior Officer	32.2%	67.8%
	Senior Officer	32.5%	67.5%
	Total	34.6%	65.4%
Statistics		Chi^2^ = 2.542, df = 3, *p* = 0.468	
Military service tenure	3–10 years	29.1%	70.9%
	11–20 years	36.5%	63.5%
	21–30 years	35.4%	64.6%
	31–40 years	33.9%	66.1%
	Total	34.5%	65.5%
Statistics		Chi^2^ = 4.658, df = 3, *p* = 0.199	
Military rank	Specialized—Technical, Equipment Operation, Operator, etc.	29.3%	70.7%
	Other Specialized	31.3%	68.7%
	Other	39.6%	60.4%
	Staff	40.8%	59.2%
	Other Staff Command (including Deputy, Command Assistant)	34.9%	65.1%
	Total	34.6%	65.4%
Statistics		Chi^2^ = 11.873, df = 4, *p* = 0.018	

## Data Availability

Data are contained within the article.
